# Jones Fracture in the National Football League

**DOI:** 10.3390/sports12010007

**Published:** 2023-12-25

**Authors:** Emily J. Luo, Albert T. Anastasio, Taylor Stauffer, Caitlin Grant, Christine J. Wu, Kevin A. Wu, Samantha Kaplan, Brian C. Lau

**Affiliations:** 1 Department of Orthopaedic Surgery, Duke University School of Medicine, Durham, NC 27710, USA; albert.anastasio@duke.edu (A.T.A.); taylor.stauffer@duke.edu (T.S.); caitlin.grant@duke.edu (C.G.); christine.j.wu@duke.edu (C.J.W.); brian.lau@duke.edu (B.C.L.); 2 Medical Center Library and Archives, Duke University, Durham, NC 27710, USA; samantha.kaplan@duke.edu

**Keywords:** Jones fracture, proximal fifth metatarsal fracture, NFL, systematic review, return to play

## Abstract

**Background:** Jones fracture, or proximal fifth metatarsal fracture, is a common injury in National Football League (NFL)-caliber athletes. Combine draft performance can greatly impact the long-term success of these athletes, and substantial emphasis has been placed on early return to play (RTP) and the minimization of post-operative complications after Jones fracture in these athletes. To date, no study has specifically described the treatment and outcomes of this injury specifically in NFL-caliber players, considering factors relevant to this unique population. Thus, the purpose of this review is to delve into Jones fracture in NFL-caliber athletes, evaluating the diagnostic, treatment, and RTP considerations. **Methods:** We searched Medline (PubMed), Embase (Elsevier), Scopus (Elsevier), and SPORTDiscus (EBSCOhost) for the concept of Jones fractures in the NFL. Using the PRISMA guidelines, a team of three reviewers conducted abstract screenings, full-text screenings, and the extraction of studies describing Jones fractures specifically in the NFL. **Results:** Of the 1911 studies identified, 6 primary retrospective studies met the inclusion and exclusion criteria. The heterogeneity of the outcome reporting precluded a meta-analysis; thus, a qualitative review of manuscripts describing Jones fracture was carried out. Classification, diagnosis, and treatment considerations, RTP statistics and outcomes, and complications were discussed. Amongst the primary studies, there were 285 Jones fractures, all athletes were able to RTP, and the average time to RTP ranged from 6 weeks to 27 weeks. For complications, with operative treatment, the refracture rate ranged from 4 to 12%, and incomplete healing ranged from 7 to 50%. RTP was 15 weeks for refractures. There were no patient-reported outcomes. **Conclusions:** The vast majority of Zone 2/3 Jones fractures are treated with IM screw fixation with or without adjunctive orthobiologics, such as bone marrow aspirate concentrate, in NFL-caliber athletes. The six major series investigating outcomes after the operative treatment of Jones fractures in NFL players reveal very positive findings overall with regard to RTP, reoperation, and career continuation.

## 1. Introduction

In the elite athlete population, the management of Jones fractures, or fractures of the proximal fifth metatarsal, can be complex, given their association with a prolonged healing time, nonunion, and the need for a swift return to play (RTP). With the mechanism of injury for Jones fractures being either repetitive weight-bearing and pivot motions on the involved foot or acute direct trauma, this fracture pattern has been observed to have a higher prevalence among elite football players, specifically among athletes in the National Football League (NFL) [1,2,3]. Moreover, fracture incidence is the highest among athletic males in the third decade of life [4], and NFL athletes are at an increased risk, especially when considering player positions and associated biomechanics. Specifically, when considering the positional movement of the defensive lineman and tight ends compared to other positions such as kickers or offensive lineman, lower extremity biomechanics are position-specific in that a higher risk for Jones fracture among the former positions has been demonstrated [5,6]. Biomedical studies suggest that differences in muscle dynamics likely contribute to the varying types of fractures experienced by players [7,8,9]. As the incidence of foot and ankle injury rates rise in elite football players, it is important to consider the management and rehabilitation of Jones fracture in an acute setting and in athletes presenting with prior injury. Previous research has demonstrated that up to 72% of collegiate players present to the NFL with a history of a foot or ankle injury [5].

The general consensus approach to Jones fracture in high-level athletes is surgical treatment with intramedullary (IM) screw fixation [10]. Despite this, non-operative approaches have been described as well [11]. While the incidence and treatment guidelines regarding Jones fractures have been studied in detail in the elite athlete population, there are only several studies that specifically evaluate this fracture pattern and RTP among athletes in the NFL. Athletes facing delayed return to play not only endure economic costs themselves, but also pose financial risks to their respective teams [12]. Our objective is to present a comprehensive overview—the first of its kind—in order to assess the current literature regarding the diagnosis, treatment guidelines, surgical management, RTP, and other considerations for NFL athletes with Jones fractures.

## 2. Materials and Methods

### 2.1. Search and Screening of the Literature

**Figure 1:**
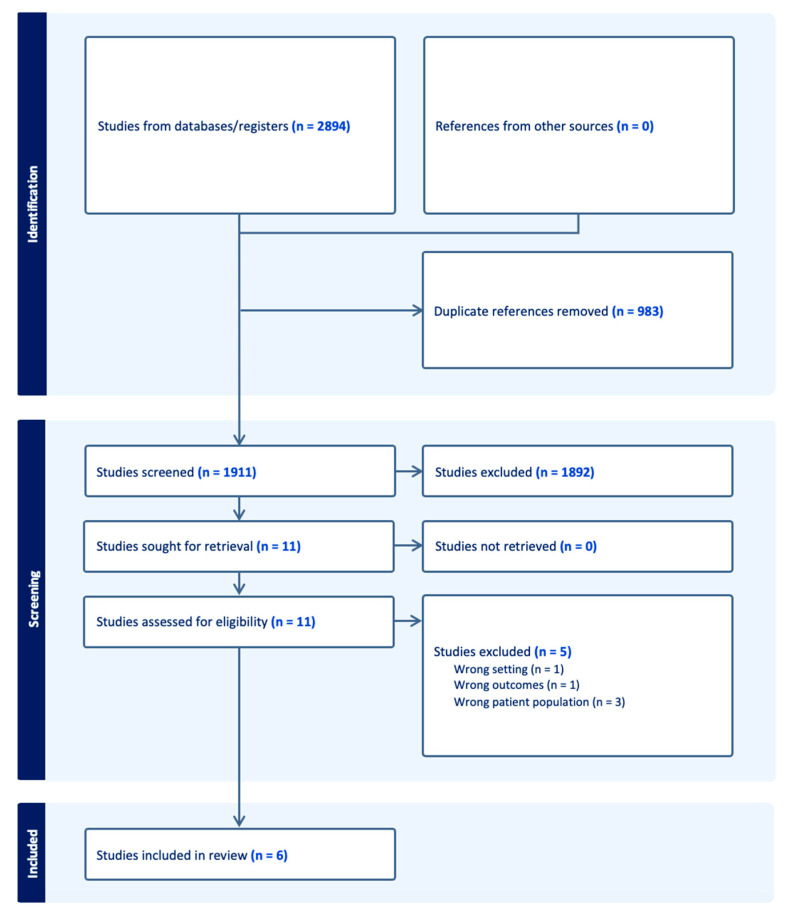
PRISMA flowchart for data extraction.

**Figure A1:**
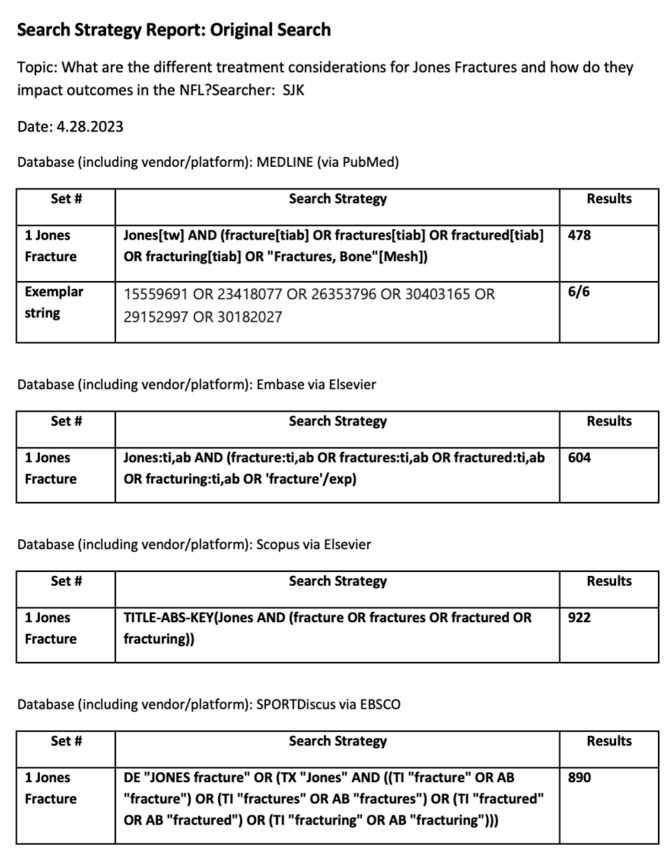
Systematic review database search strategy.

This study followed the PRISMA guidelines (Supplementary Material File) [13]. We searched Medline (PubMed), Embase (Elsevier), Scopus (Elsevier), and SPORTDiscus (EBSCOhost) on 3 February 2023 using a combination of keywords and database-specific subject headings for the concept of Jones fractures. The full, reproducible search strategies for all included databases are in the Appendix (Figure A1). The searches yielded a total of 2894 citations. All citations were imported into an Endnote library for storage before being sent to Covidence, a systematic review screening software (Veritas Health, Melbourne, Australia); Covidence detected and automatically removed 983 duplicates, leaving 1911 unique citations for screening (Figure 1). This review was not registered.

Abstracts and then full texts were independently screened by three reviewers with the inclusion criteria being primary studies describing treatment, return to play outcomes, and guidelines for patients in the NFL who have had a Jones fracture. Review articles, case reports, non-English articles, and opinion pieces were excluded. A total of 6 primary studies were found to fit all inclusion and exclusion criteria. All voting disagreements at abstract and full-text levels were resolved with discussion. Data extraction was performed manually, with one reviewer extracting data for each article. Data extracted from each article included citation details, author, year, sample size, treatment, RTP outcomes, complications, position data, revision outcomes, and study endpoints, which are summarized in Table 1. 

Due to the heterogeneity of data and low number of primary studies, a qualitative review rather than a meta-analysis was performed. We hypothesized that treatment would be primarily operative with strong return-to-sport outcomes and few complications. 

### 2.2. Risk of Bias and Quality Assessment

All studies were assessed for bias and study quality using the Methodological Index for Nonrandomized studies (MINORS) criteria [16]. The MINORS criteria consist of a 12-item checklist, with each criterion assigned a score of 0 (not reported), 1 (inadequately reported), or 2 (adequately reported). Noncomparative studies can have a total score of up to 16 points, while comparative studies can have a maximum of 24 points. The risk of bias and quality assessment can be found in Appendix A, Table A1. 

## 3. Results

### 3.1. Classification

**Figure 2:**
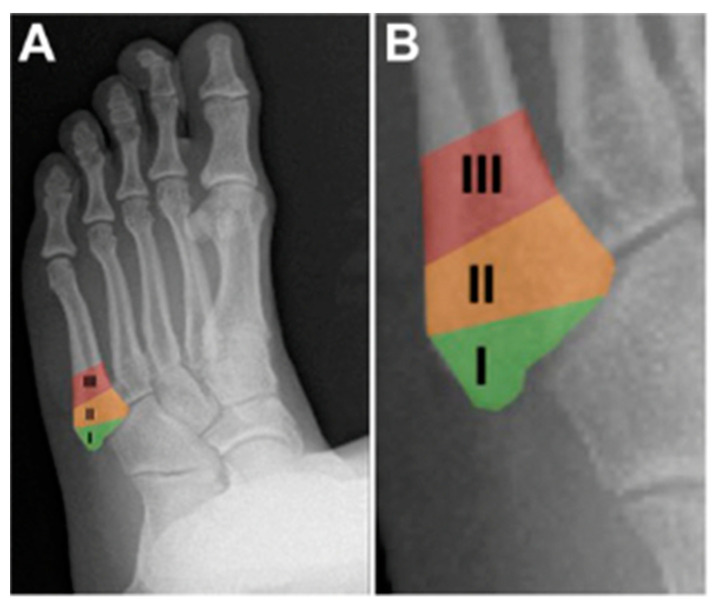
Normal radiograph of a left foot. (**A**) The 3 zones: Zone 1 (tuberosity avulsion fracture), Zone 2 (Jones fracture), and Zone 3 (proximal diaphyseal fracture (stress fracture)). (**B**) Closer view of the zones [5].

Jones fractures occur approximately 1.5 cm distal to the styloid of the fifth metatarsal at the metaphyseal diaphyseal junction, otherwise defined as where the fourth and fifth metatarsals articulate [1]. The base of the fifth metatarsal contains a vascular watershed area with retrograde blood flow, resulting in a heightened risk of poor healing. There have been multiple classification systems devised since the recognition of this injury. Briefly, fractures of the fifth metatarsal were first classified by location and morphology in 1960 by Stewart [17]. Another classification system based on radiographs was then developed by Torg et al., and then again by Dameron in 1975 after a large clinical series investigation [18,19]. By 1993, Lawrence et al. reported fractures by anatomic location, which were later organized into zones in 1995 by Dameron [20,21]. To date, this zone system is the most widely used, with Zone 1 being the most proximal fracture type and occurring at the peroneus tendon insertion and the metatarso-cuboid joint. Zone 2, a true Jones fracture, occurs at the metaphyseal–diaphyseal junction and extends into the fourth and fifth metatarsal articulation or intermetatarsal ligament. Lastly, Zone 3 fractures occur distal to the intermetatarsal articulation (Figure 2). Some authors have advocated for the consideration of Zone 2/3 fractures as similar injuries, both exhibiting high rates of nonunion and preferably treated surgically, while Zone 1 injuries can be treated non-operatively [22].

### 3.2. Diagnosis

Due to the proposed vascular watershed at the proximal fifth metatarsal and the biomechanical stresses placed upon the region from the transition from metaphyseal to diaphyseal, the risk for nonunion, persistent pain, and refracture in Jones fracture is high. Cavus and skew foot alignment also increase stress in this region. Thus, a swift diagnosis is a key element in the successful management of this injury [23]. Providers should ask for details regarding the injury mechanism, with adduction force applied to the forefoot with a plantar-flexed ankle being a common mechanism for Jones fracture [20]. The mechanism of injury is similar to a lateral ankle sprain, and therefore, the base of the fifth metatarsal should be palpated for tenderness for any inversion ankle injury.

Robust ligamentous, fascial, and tendinous attachments resist significant displacement. In athletic motions, these injuries frequently occur in a sudden change in direction with the heel off the ground and missteps on the lateral midfoot. In overuse injuries, athletes may have pain in the lateral foot after excessive activity or change in activity level, and symptoms occasionally may be related to changes in shoes or cleats [23]. Differential diagnosis includes peroneus brevis tendinitis, cuboid fracture, os peroneum, and tarsometatarsal joint sprain. Patients often present with point tenderness over the proximal area of the fifth metatarsal with lateral foot swelling in the setting of the acute injury [14].

**Figure 3:**
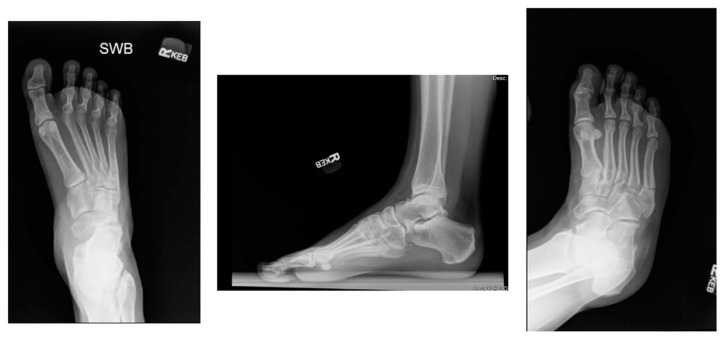
Weight-bearing AP, lateral, and oblique radiographs of an acute, non-displaced Jones fracture before ORIF in a 21-year-old male.

An evaluation should include an assessment of the hindfoot alignment, ankle stability, and peroneal strength against resisted eversion [24,25,26]. The initial imaging in the workup should include standard radiographs of the foot, including anteroposterior, lateral, and oblique views [23]. Weight-bearing radiographs may be helpful if tolerated. Clinical images of Jones fractures are demonstrated in Figure 3.

In patients with nondiagnostic radiographs, magnetic resonance imaging (MRI) aids in detecting stress reactions or soft tissue abnormalities. Computed tomography (CT) scans may be utilized to assess healing after fracture stabilization. 

### 3.3. Treatment Considerations

#### 3.3.1. Non-Operative Treatment

The non-operative treatment of a Jones fracture for the general population typically consists of elevation for swelling control, and then weight bearing, as tolerated, with a stiff-soled orthosis [27]. Although non-operative management in some instances of Jones fractures may be successful, the healing time and RTP are critical factors in decision making for elite athletes and NFL players in particular. The work by Torg et al. first described immobilization with a non-weight-bearing boot as the primary treatment of choice [18]. These authors asserted that with enough time, union would occur in almost all patients if vigorous activity was avoided. However, they also note that in athletes who desire to return to competition and full activity, prolonged immobilization may not be acceptable [18]. For patients who are deemed appropriate for non-operative management, prolonged immobilization is the hallmark of conservative management, with the utilization of a CAM boot for 6–8 weeks and serial radiographs to evaluate healing [3,27]. Although conservative management continues to be used in some cases, delayed union rates as high as 66%, nonunion rates of up to 28%, and refracture rates of up to 33% have been reported with non-operative treatment [3]. Newer case series in other elite athlete populations have looked into conservative treatment using platelet-rich plasma (PRP) as a potential non-surgical alternative; however, these studies have yet to be replicated in American athletes [28].

#### 3.3.2. Surgical Treatment Techniques

The studies of Jones fracture in the NFL report on Zone 2/3 fractures, and there are no studies of non-operative treatment of Zone 1 fractures in the NFL. In studies of the general population, the conservative management of Zone 1 fractures has impressive outcomes, with nearly all patients achieving radiographic healing [29]. 

A recent systematic review and meta-analysis of 22 studies of athletes (not specifically NFL players) with Jones fractures revealed a higher rate of RTP with a shorter duration away from sport, a higher rate of union, a shorter time to union, and improved functional outcomes with operative management compared to non-operative treatment [30]. Thus, operative intervention is typically preferred for elite athletes for both displaced and non-displaced Zone 2/3 fractures. 

**Figure 4:**
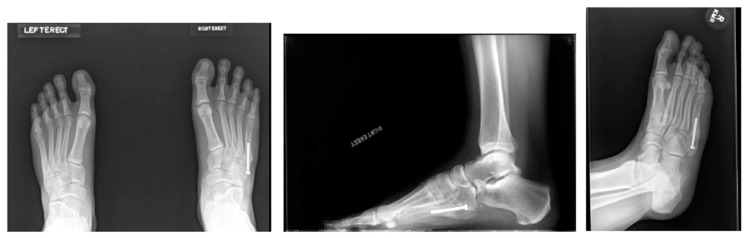
Weight-bearing AP, lateral, and oblique radiographs of Patient 1 (21-year-old male) after IM screw fixation.

Surgical management with IM screw fixation is currently the most accepted method of treatment for Jones fractures for NFL players and other elite athletes. Between 2009 and 2015, of all NFL Combine athletes, 72 athletes had previous Jones fractures, all of whom were treated with an IM screw [5]. Hsu et al. describe their method of percutaneous IM screw fixation with a canulated drilling entry point at the proximal third of the fifth metatarsal. They note technique specifications, such as utilizing the largest possible screw that does not abut the lateral cortex with a low-profile head. The IM canal of the fifth metatarsal has been measured as greater than 4.5 mm in 81% males, and therefore, screw sizes of at least 4.5, 5.5, and 6.5 mm have been recommended in order to reduce fatigue failure and complications [6]. With screw fixation, the importance of avoiding straightening the natural curve of the fifth metatarsal has also been described to prevent lateral gapping and an increased nonunion risk [3]. For career football athletes, Hsu et al. augment the fracture site with bone marrow aspirate concentrate (BMAC) and demineralized bone matrix (DBM). Using this method in a cohort of 25 NFL players, 100% of the athletes were able to RTP at an average of 9.5 weeks with a 7.5% reoperation rate [3,14]. Clinical radiographs of IM screw fixation in a 21-year-old male (Figure 3) are demonstrated in Figure 4**.**

Plantar plating, although less common than IM screw fixation, has also been utilized for Jones fracture fixation in elite athletes. This method of fixation has been described to directly resist tensile forces and gap formation as well as contour to the bone, which is thought to decrease the risk of nonunion and refracture rates that persist with IM screw fixation [31]. In a small cohort of eight elite athletes, including four NFL players with primary fracture and refracture of the fifth metatarsal, all patients demonstrated clinical and radiographic union at an average of 6.5 weeks post-surgery, and full activity was achieved at an average of 12 weeks after surgery [31]. While seemingly successful with regard to the union rate and RTP, some authors raised concerns with plantar plating for high complication rates, such as peroneal tendon irritation and hardware prominence [32]. Although promising, plantar plating is primarily used in the revision setting, and therefore, a direct comparison is not possible. Larger studies examining the efficacy of plantar plating as well as comparative work examining plating versus IM screw fixation outcomes in Jones fractures is still needed. 

### 3.4. Rehabilitation and Return to Play

Although surgical protocols for the operative management of Jones fractures in NFL players is relatively consistent, post-operative management and rehabilitation is more variable. An individualized approach for in-season athletes after IM screw fixation with 1 to 2 weeks of non-weight bearing followed by 2 to 4 weeks of toe-touch weight bearing in a tall CAM boot is described in [3,14]. After 4 weeks, these authors described an incremental increase in exercise intensity with stationary biking, pool therapy, and if nontender, running in a protective shoe. During the post-operative period, Hsu et al. also utilized adjuvant low-intensity pulsed ultrasound bone stimulators over the fracture site. With this individualized clinical approach, Hsu and Lareau et al. described an average RTP of 6–10 weeks with radiographic union sometimes not evident until the 12-to-16-week mark. 

Other important RTP aids, whether treating the patient non-operatively or operatively, include custom orthosis with turf toe plates, a cushioned lateral column, and a lateral heel wedge if the athlete’s varus deformity is present [3,14]. A first-ray recess may also be useful if the athlete’s varus is forefoot-driven.

### 3.5. Complications

While the post-operative outcomes for Jones fracture repair are typically successful, potential complications can be divided into general complications and operation-related complications. General complications may include refracture, malunion, and delayed union/nonunion [33]. For refractures or nonunions, the overall incidence is approximately 12% for IM screw fixation and typically requires revision surgery, which may include an iliac crest bone graft with repeat screw placement [6,14,32]. In elite athletes, however, the rate of refracture can be as high as 30% for IM screw fixation and 10.5% for plate fixation [34,35]. Operation-related complications include wound infection, delayed wound healing, sural nerve injury, hardware failure, refracture, malunion, delayed union/nonunion, a prominence of screwhead, chronic low-level pain, and iatrogenic fracture [32,36]. 

Amongst the NFL athletes studied, the incidence of operation-related complications was essentially zero [14,36]. Instead, the primary complications affecting NFL athletes include either refracture or incomplete healing [2,6,14]. The refracture rates range from 7.5 to 12%, and the rate of incomplete bony union is approximately 8% [6,14]. The overall incomplete healing rates were as high as 50%, with the plantar cortex showing the least healing [5]. While the cause of refractures in NFL athletes requires further study, one group showed that the return to full activity prior to complete radiographic union was the best predictor of failure following Jones fracture fixation [37]. Other studies have reported, however, cases of refracture in professional football athletes despite full clinical/radiographic healing and RTP clearance [14,38]. Small diameter screws (less than 4.5 mm) have also been associated with increased rates of both refracture and delayed union [39]. Other causes of delayed return to play or nonunion can be stratified into patient-specific parameters (smoking status, diabetes, peripheral vascular disease, vitamin D/calcium deficiency, hormonal deficiency, and increased age), injury-specific parameters (Zone 2/3 fractures and open fractures), and medications (non-steroidal anti-inflammatory drugs (NSAIDs), synthetic glucocorticoids, and chemotherapy agents) [32,40,41,42,43,44,45]. 

### 3.6. NFL RTP and Outcomes

#### 3.6.1. Treatment Methodology

Of the six retrospective studies that analyzed NFL athlete outcomes after Jones fracture, four of these studies reported on Combine athletes, and two studies investigated outcomes in professional players (Table 2). Amongst the four studies of NFL Combine athletes, all studies, apart from that by Low et al., reported operative treatment with IM screw fixation for all Jones fractures [11]. They also showed that from 1988 to 1998, 35% of fractures were treated operatively. By 1997, however, 68% of the fractures were treated with surgery. Tu et al. also found that players with refracture required revision surgery with an iliac crest bone graft in addition to repeat screw placement [6]. 

In professional NFL players, Lareau et al. noted that all players were treated with IM screw fixation as well as the injection of iliac crest bone marrow aspirate (BMA) and DBM [14]. The treatment methodology was not available for Singh et al., although all included athletes were treated with surgery. Lareau et al. reported an average of 8.5 days between the injury and surgery [14]. 

#### 3.6.2. RTP Outcomes

Amongst NFL athletes and prospects, the RTP rate after Jones fracture is essentially 100% [5,14]. In NFL athletes, there was a significant discrepancy in the time to RTP, with a range from 8.7 to 27 weeks [14,15]. For NFL prospects at Combine, RTP is also not as well defined across the literature. Low et al. reported that 86% of athletes were able to RTP in 6–12 weeks [11]. Spang et al. reported that most athletes did not miss any collegiate games due to Jones fractures (N = 62/67, 93%), perhaps due to athletes receiving surgery in the off season [5]. Tu et al. found that the average time from surgery to Combine was 27 months (SD 22.3 months), but this measurement does not adequately capture the average time for collegiate athletes to RTP post-Jones fracture repair [6]. 

#### 3.6.3. Draft Prospective and NFL Performance

Between the four studies on NFL Combine athletes, there were some discrepancies in the draft prospects and NFL performance outcomes. While Carreira et al. and Tu et al. both reported no statistically significant differences in the draft prospects for athletes with a prior Jones fracture, Spang et al. found that the Jones fracture group had a worser mean overall draft pick compared to the control group (125.4 vs. 99.0, respectively, *p* < 0.03) [5]. Additionally, defensive linemen and running backs with a previous Jones fracture had significantly lower NFL fantasy scores. All positions, aside from quarterbacks and linebackers, had significantly lower snap percentages in their first 3 years in the NFL. The fracture group also had fewer number of games played. When analyzing performance in relation to healing measured by cumulative CT score distribution, Spang et al. found that players with little or no healing (score < 1) started in fewer total games (2.7 +/− 2.5), and players with an anatomically fully healed fracture played two or more NFL seasons [5]. Carreira et al. found that the number of players who played less than 10 games in the NFL was significantly higher in the fracture group compared to the control [2]. However, similar to Tu et al., there was no statistically significant difference in the total number of NFL games played. Tu et al. also did not find a statistical difference in starting NFL games or the number of games played between the fracture and control groups [6]. 

Unlike NFL Combine athletes, professional NFL players with Jones fractures had overall favorable performance outcomes post-fixation. Lareau et al. reported that amongst the 25 NFL players who underwent operative fixation and had a Zone 2/3 fracture, 80% of players were still playing by the end of the season, the average number of seasons played post-injury was 2.7 seasons, and two athletes were selected as Pro-Bowl players after surgery [14]. Similarly, Singh et al. reported an average post-injury career of 3.8 years [15]. However, the group noted a decrease in performance in the three seasons post-Jones fracture surgery when using previously validated performance scores. 

## 4. Discussion

### 4.1. Overview

Our review is the first of its kind to evaluate the treatment of Jones fracture in the NFL across the literature. An overview of the key points can be found in Table 2. With the 2004 study from Low and colleagues demonstrating significantly increased nonunion rates with non-operative treatment compared to operative treatment, there was a paradigm shift with all NFL studies thereafter utilizing operative treatment. Operative treatment nearly universally consists of IM screw fixation with or without adjuncts such as BMAC. The post-operative protocol is much more varied, although newer technologies such as adjuvant low-intensity pulse ultrasound bone stimulation could potentially improve RTP timing. It should, however, be noted that there is a discrepancy in treatment, and thus in outcomes, between professional NFL players and prospective athletes at the Combine. Only NFL players were shown to receive IM screw fixation with the injection of BMA and DBM. In contrast, only NFL Combine players with refractures received the full gamut of orthobiologic adjuncts plus operative management [6]. This difference in treatment may reflect the differences in outcomes shown between the two levels, with Lareau et al. reporting better performance for players currently in the NFL compared to other studies of only Combine athletes. While this could point to adjunctives such as BMAC having the potential to provide better player outcomes, future investigations should seek to directly compare isolated IM screw fixation versus IM screw fixation plus BMA plus DBM or other orthobiologics and how the cumulative CT score distribution and performance outcomes are impacted in NFL athletes versus NFL Combine players. Additional orthobiologics used as conservative treatments such as PRP should also be explored for NFL athletes, as they have shown to allow return to training in 43–50 days in case series from other professional sports leagues [28]. The utility of other bone autografts aside from the iliac crest may also be an area of study, as calcaneal autografts have shown to be the most popular for other elite athlete populations [10].

In terms of NFL player outcomes, there were significant differences between the studies regarding complication rates, draft prospects, number of games started, and number of games played. The discrepancies in the study outcomes could be due to differences in the sample size. Additionally, Spang et al. was the only study to stratify outcomes based on the cumulative CT score, which may have provided increased granularity to discern statistical differences. Between the NFL studies, it should be noted that Singh et al. compared NFL athletes to other professional athletes in the National Basketball Association, Major League Baseball, and National Hockey League, which may have affected the statistical analyses and significance. 

In addition to differences in the reported outcomes between prospective and professional NFL athletes, the studies also showed position discrepancies. Spang et al. and Tu et al. found that amongst NFL Combine athletes, tight ends were at a higher risk of experiencing Jones fractures. Low et al. showed that offensive lineman and defensive lineman had the greatest percentage of fracture. Amongst NFL athletes, more than 50% of the players included in the Lareau et al. study were wide receivers or linebackers. More studies are needed to analyze the biomechanics behind these position discrepancies in Jones fracture incidence. 

### 4.2. Strengths and Limitations

In regard to the study limitations, our study was limited by the small number of studies on this topic and necessitated a qualitative review. Additionally, none of our included studies specifically excluded other concurrent injuries in addition to Jones fractures, which may have affected the statistical significance and findings. The six studies also differed in terms of defining a control group. Moreover, some of the studies either did not have a control group or had a poorly defined control group consisting of various other foot injuries. Lastly, there were several differences in the types of outcomes reported by the four studies, making it difficult to draw conclusions across the literature on which findings are statistically significant and replicable. The strength of our study is that this review provides, for the first time, a comprehensive overview of Jones fracture in the NFL, starting from diagnosis and injury classification to player outcomes. With these fractures being among the most common foot and ankle injuries in American football, this review may serve as a guide for providers at all levels in the early recognition, work-up, successful management, and rehabilitation of NFL athletes presenting with this pathology. 

## 5. Conclusions

In summary, Jones fracture is a common injury in NFL-caliber athletes. The emphasis on treatment remains IM screw fixation with or without adjunctive orthobiologics, such as BMAC. The post-operative protocol differs substantially across the literature. The six major series investigating outcomes after the operative treatment of Jones fractures in NFL players reveal positive findings with regard to RTP, reoperation, and career continuation, but also heterogeneity in the complication rates. Future research should aim to explore the benefits of orthobiologics used at the time of surgery and should rigorously compare various post-operative and RTP protocols for this common injury.

## Figures and Tables

**Table 1:** (**a**). Study characteristics of the six primary series evaluating the outcomes of NFL-caliber players being treated for Jones fracture. NR = not reported. BMAC = bone marrow aspirate concentrate. DBM = demineralized bone matrix. (**b**) Study endpoints of the six primary series. CT = computed tomography.

(a)									
Author, Year	Study Group (Year)	Sample Size	Treatment	RTP Outcomes	Overall Outcomes	Position Data	Complications	Revision Outcomes	Quality Assessment
Low, 2004 [11]	NFL Combine (1988–2002)	86 Jones fractures	Non-operative, operative (IM screw)	86% RTP in 6–12 weeks	Operative: 43/46 fractures healed without problems,	Higher incidence of fractures in offensive vs. defensive players (61% vs. 39%). Most common positions: offensive, defensive linemen	Non-operative: 20% nonunion Operative: 7% nonunion	NR	12
Carreira, 2013 [2]	NFL Combine (2004–2009)	45 Jones fractures	Operative (IM Screw)	NR	no significant differences in being drafted, increased number of players who played <10 games in NFL	NR	NR	NR	14
Lareau, 2016 [14]	NFL athletes (2004–2014)	25 Jones fractures	Operative (IM screw + BMAC + DBM)	25/25 (100%) RTP, average 8.7 weeks	80% still playing by end of season, average of 2.7 season played post-injury, 2 pro-bowl players after surgery	NR	12% refracture + required revision surgery	NR	12
Singh, 2018 [15]	NFL athletes (1986–2016)	17 Jones Fractures	Operative	RTP median = 189 days (27 weeks)	Drop in performance level after surgery, mean average career after surgery: 3.8 +/− 3.7 years	NR	No refractures	NR	10
Spang, 2018 [5]	NFL Combine (2009–2015)	72 Jones fractures	Operative (IM Screw)	67/72 (93%) athletes did not miss any collegiate games	46/72 players >2 years in NFL, higher mean overall draft pick number, more athletes undrafted, lower fantasy scores, lower snap percentages	Tight ends more likely to have Jones fractures, defensive backs and running backs have lower risk	50% showed incomplete healing at Combine	NR	12
Tu, 2018 [6]	NFL Combine (2012–2015)	40 Jones Fractures	Operative (IM screw +/− BMAC + DBM)	Average time from surgery to Combine: 27 +/− 22.3 months	No limitations in strength/ROM, no increased risk of going undrafted, playing, or starting in NFL games	Highest prevalence of fracture repair in defensive lineman, highest rate of fracture in tight ends	7.5% re-fracture rate requiring revision surgery, incomplete bony union in 8% fractures	RTP 15 weeks for re-fractures, no association between RTP and refracture rate, 100% had CT evidence of union	11
**(b)**									
Author, Year		Study Conclusion							
Low, 2004 [11]		IM screw fixation of Jones fracture is the treatment of choice for elite college and professional football athletes.							
Carreira, 2013 [2]		No statistically significant difference in NFL participation after Jones fracture, though there was a trend towards decreased participation.							
Lareau, 2016 [14]		IM screw fixation and aggressive rehabilitation protocol allowed for early RTP and low refracture rate in NFL athletes. All players returned to play.							
Singh, 2018 [15]		Regardless of sport or fracture location, athletes returned to play at a high rate after foot fracture location and had excellent post-operative performance levels.							
Spang, 2018 [5]		On CT scan, 50% of all NFL Combine players with a previous Jones fracture had incomplete healing. Players with fractures had lower position-specific performance scores over the first 2 years of career. Players with lower CT scores started fewer games and were drafted later.							
Tu, 2018 [6]		Players with Jones fracture repair did not have a significantly increased risk of going undrafted or having decreased participation in their first NFL season.							

**Table 2:** Overview of Jones fracture in the NFL.

Jones Fracture in the NFL	
Incidence	17.8% of all foot fractures
Average time to RTP	Range: 6–27 weeks
Incidence of complications	Operative management: refracture: 4–12%; incomplete healing: 7–50% Non-operative management: 20% nonunion
Main treatment modality	Intramedullary screw fixation; may also include BMAC + DBM
Rehabilitation	Non-weight bearing → toe-touch weight bearing → increase exercise intensity incrementally +/− low intensity pulsed ultrasound bone stimulator

## Data Availability

No new data were created for this research.

## Supplementary Materials

The following supporting information can be downloaded at: https://www.mdpi.com/article/10.3390/sports12010007/s1, Supplementary File S1: PRISMA 2020 Checklist.

## Appendix A

**Table A1:** Risk of bias and quality assessment.

Nonrandomized Cohort Studies						
MINORS	Tu 2018 [6]	Spang 2018 [5]	Singh 2018 [15]	Lareau 2016 [14]	Carreira 2013 [2]	Low 2004 [11]
A clearly stated aim	2	2	2	2	2	2
Inclusion of consecutive patients	2	2	1	2	2	2
Prospective collection of data	0	0	0	0	2	1
Endpoints appropriate to the aim of the study	2	2	2	2	2	2
Unbiased assessment of study endpoint	1	2	1	2	2	1
Follow-up period appropriate to the aim of the study	2	2	2	2	2	2
Loss to follow up less than 5%	2	2	2	2	2	2
Prospective calculation of the study size	0	0	0	0	0	0
Total	11	12	10	12	14	12

## References

[1] Sammarco G.J. (1993). The Jones fracture. Instr. Course Lect..

[2] Carreira D.S., Sandilands S.M. (2013). Radiographic Factors and Effect of Fifth Metatarsal Jones and Diaphyseal Stress Fractures on Participation in the NFL. Foot Ankle Int..

[3] Hsu A.R., Anderson R.B. (2016). Foot and Ankle Injuries in American Football. Am. J. Orthop..

[4] Petrisor B.A., Ekrol I., Court-Brown C. (2006). The epidemiology of metatarsal fractures. Foot Ankle Int..

[5] Spang R.C., Haber D.B., Beaulieu-Jones B.R., Stupay K.L., Sanchez G., Sanchez A., Murphy C.P., Whalen J.M., Van Allen J.J., Price M.D. (2018). Jones Fractures Identified at the National Football League Scouting Combine: Assessment of Prognostic Factors, Computed Tomography Findings, and Initial Career Performance. Orthop. J. Sports Med..

[6] Tu L.A., Knapik D.M., Sheehan J., Salata M.J., Voos J.E. (2018). Prevalence of Jones Fracture Repair and Impact on Short-Term NFL Participation. Foot Ankle Int..

[7] Hoffman J.R. (2008). The applied physiology of American football. Int. J. Sports Physiol. Perform..

[8] Solarino G., Bortone I., Vicenti G., Bizzoca D., Coviello M., Maccagnano G., Moretti B., D’Angelo F. (2021). Role of biomechanical assessment in rotator cuff tear repair: Arthroscopic vs mini-open approach. World J. Orthop..

[9] Escamilla R.F., Andrews J.R. (2009). Shoulder muscle recruitment patterns and related biomechanics during upper extremity sports. Sports Med..

[10] Goodloe J.B., Cregar W.M., Caughman A., Bailey E.P., Barfield W.R., Gross C.E. (2021). Surgical Management of Proximal Fifth Metatarsal Fractures in Elite Athletes: A Systematic Review. Orthop. J. Sports Med..

[11] Low K., Noblin J.D., Browne J.E., Barnthouse C.D., Scott A.R. (2004). Jones fractures in the elite football player. J. Surg. Orthop. Adv..

[12] Abed V., Fine R., Fine R., Hawk G.S., Conley C., Jacobs C., Stone A.V. (2023). Return to Play, Performance, and Economic Analysis of National Football League Players After Lisfranc Injury. Orthop. J. Sports Med..

[13] Page M.J., McKenzie J.E., Bossuyt P.M., Boutron I., Hoffmann T.C., Mulrow C.D., Shamseer L., Tetzlaff J.M., Akl E.A., Brennan S.E. (2021). The PRISMA 2020 statement: An updated guideline for reporting systematic reviews. BMJ.

[14] Lareau C.R., Hsu A.R., Anderson R.B. (2016). Return to Play in National Football League Players After Operative Jones Fracture Treatment. Foot Ankle Int..

[15] Singh S.K., Larkin K.E., Kadakia A.R., Hsu W.K. (2018). Risk Factors for Reoperation and Performance-Based Outcomes After Operative Fixation of Foot Fractures in the Professional Athlete: A Cross-Sport Analysis. Sports Health.

[16] Slim K., Nini E., Forestier D., Kwiatkowski F., Panis Y., Chipponi J. (2003). Methodological index for non-randomized studies (minors): Development and validation of a new instrument. ANZ J. Surg..

[17] Stewart I.M. (1960). Jones’s Fracture: Fracture of Base of Fifth Metatarsal. Clin. Orthop. Relat. Res..

[18] Torg J.S., Balduini F.C., Zelko R.R., Pavlov H., Peff T.C., Das M. (1984). Fractures of the base of the fifth metatarsal distal to the tuberosity. Classification and guidelines for non-surgical and surgical management. J. Bone Joint Surg. Am..

[19] Dameron T.B. (1975). Fractures and anatomical variations of the proximal portion of the fifth metatarsal. J. Bone Joint Surg. Am..

[20] Lawrence S.J., Botte M.J. (1993). Jones’ fractures and related fractures of the proximal fifth metatarsal. Foot Ankle.

[21] Dameron T.B. (1995). Fractures of the Proximal Fifth Metatarsal: Selecting the Best Treatment Option. J. Am. Acad. Orthop. Surg..

[22] Michalski M.P., Ingall E.M., Kwon J.Y., Chiodo C.P. (2022). Reliability of Fifth Metatarsal Base Fracture Classifications and Current Management. Foot Ankle Int..

[23] Metzl J.A., Bowers M.W., Anderson R.B. (2022). Fifth Metatarsal Jones Fractures: Diagnosis and Treatment. JAAOS-J. Am. Acad. Orthop. Surg..

[24] Riegger M., Muller J., Giampietro A., Saporito A., Filardo G., Treglia G., Guidi M., Candrian C. (2022). Forefoot Adduction, Hindfoot Varus or Pes Cavus: Risk Factors for Fifth Metatarsal Fractures and Jones Fractures? A Systematic Review and Meta-Analysis. J. Foot Ankle Surg..

[25] Raikin S.M., Slenker N., Ratigan B. (2008). The association of a varus hindfoot and fracture of the fifth metatarsal metaphyseal-diaphyseal junction: The Jones fracture. Am. J. Sports Med..

[26] Fleischer A.E., Stack R., Klein E.E., Baker J.R., Weil L., Weil L.S. (2017). Forefoot Adduction Is a Risk Factor for Jones Fracture. J. Foot Ankle Surg..

[27] Khairul Faizi Mohammad B.Y., Nousiainen M.T., Buckley R. Nonoperative Treatment—Proximal articular fractures of the 5th metatarsal (Jones fracture). AO Surgery Reference.

[28] Bezuglov E., Zholinsky A., Chernov G., Khaitin V., Goncharov E., Waskiewicz Z., Barskova E., Lazarev A. (2022). Conservative Treatment of the Fifth Metatarsal Bone Fractures in Professional Football Players Using Platelet-Rich Plasma. Foot Ankle Spec..

[29] Nishikawa D.R.C., Aires Duarte F., Saito G.H., Bang K.E., Monteiro A.C., Prado M.P., de Cesar Netto C. (2020). Treatment of Zone 1 Fractures of the Proximal Fifth Metatarsal With CAM-Walker Boot vs Hard-Soled Shoes. Foot Ankle Int..

[30] Attia A.K., Taha T., Kong G., Alhammoud A., Mahmoud K., Myerson M. (2021). Return to Play and Fracture Union After the Surgical Management of Jones Fractures in Athletes: A Systematic Review and Meta-analysis. Am. J. Sports Med..

[31] Bernstein D.T., Mitchell R.J., McCulloch P.C., Harris J.D., Varner K.E. (2018). Treatment of Proximal Fifth Metatarsal Fractures and Refractures With Plantar Plating in Elite Athletes. Foot Ankle Int..

[32] Chloros G.D., Kakos C.D., Tastsidis I.K., Giannoudis V.P., Panteli M., Giannoudis P.V. (2022). Fifth metatarsal fractures: An update on management, complications, and outcomes. EFORT Open Rev..

[33] Cheung C.N., Lui T.H. (2016). Proximal Fifth Metatarsal Fractures: Anatomy, Classification, Treatment and Complications. Arch. Trauma. Res..

[34] O’Malley M., DeSandis B., Allen A., Levitsky M., O’Malley Q., Williams R. (2016). Operative Treatment of Fifth Metatarsal Jones Fractures (Zones II and III) in the NBA. Foot Ankle Int..

[35] Young K.W., Kim J.S., Lee H.S., Jegal H., Park Y.U., Lee K.T. (2020). Operative Results of Plantar Plating for Fifth Metatarsal Stress Fracture. Foot Ankle Int..

[36] Albloushi M., Alshanqiti A., Qasem M., Abitbol A., Gregory T. (2021). Jones type fifth metatarsal fracture fixation in athletes: A review and current concept. World J. Orthop..

[37] Larson C.M., Almekinders L.C., Taft T.N., Garrett W.E. (2002). Intramedullary screw fixation of Jones fractures. Analysis of failure. Am. J. Sports Med..

[38] Wright R.W., Fischer D.A., Shively R.A., Heidt R.S., Nuber G.W. (2000). Refracture of proximal fifth metatarsal (Jones) fracture after intramedullary screw fixation in athletes. Am. J. Sports Med..

[39] Smith J.W., Arnoczky S.P., Hersh A. (1992). The intraosseous blood supply of the fifth metatarsal: Implications for proximal fracture healing. Foot Ankle.

[40] Rosenberg G.A., Sferra J.J. (2000). Treatment strategies for acute fractures and nonunions of the proximal fifth metatarsal. J. Am. Acad. Orthop. Surg..

[41] Nolte P., Anderson R., Strauss E., Wang Z., Hu L., Xu Z., Steen R.G. (2016). Heal rate of metatarsal fractures: A propensity-matching study of patients treated with low-intensity pulsed ultrasound (LIPUS) vs. surgical and other treatments. Injury.

[42] Blum A., Zarqh O., Peleg A., Sirchan R., Blum N., Salameh Y., Ganaem M. (2012). Vascular inflammation and endothelial dysfunction in fracture healing. Am. J. Orthop..

[43] Jiao H., Xiao E., Graves D.T. (2015). Diabetes and Its Effect on Bone and Fracture Healing. Curr. Osteoporos. Rep..

[44] Karlamangla A.S., Burnett-Bowie S.M., Crandall C.J. (2018). Bone Health During the Menopause Transition and Beyond. Obstet. Gynecol. Clin. N. Am..

[45] Brinker M.R., O’Connor D.P., Monla Y.T., Earthman T.P. (2007). Metabolic and endocrine abnormalities in patients with nonunions. J. Orthop. Trauma..

